# Image processing based modeling for *Rosa roxburghii* fruits mass and volume estimation

**DOI:** 10.1038/s41598-024-65321-9

**Published:** 2024-07-05

**Authors:** Zhiping Xie, Junhao Wang, Yufei Yang, Peixuan Mao, Jialing Guo, Manyu Sun

**Affiliations:** School of Mechanical & Electrical Engineering, Guizhou Normal University, Guiyang, China

**Keywords:** Image measurement, *Rosa roxburghii*, Physical characteristic, Estimated modeling, Grading, Assay systems, Image processing, Classification and taxonomy

## Abstract

The mass and volume of *Rosa roxburghii* fruits are essential for fruit grading and consumer selection. Physical characteristics such as dimension, projected area, mass, and volume are interrelated. Image-based mass and volume estimation facilitates the automation of fruit grading, which can replace time-consuming and laborious manual grading. In this study, image processing techniques were used to extract fruit dimensions and projected areas, and univariate (linear, quadratic, exponential, and power) and multivariate regression models were used to estimate the mass and volume of *Rosa roxburghii* fruits. The results showed that the quadratic model based on the criterion projected area (*CPA*) estimated the best mass (R^2^ = 0.981) with an accuracy of 99.27%, and the equation is M = 0.280 + 0.940*CPA* + 0.071*CPA*^2^. The multivariate regression model based on three projected areas (*PA*_1_, *PA*_2_, and *PA*_3_) estimated the best volume (R^2^ = 0.898) with an accuracy of 98.24%, and the equation is V = − 8.467 + 0.657*PA*_1_ + 1.294*PA*_2_ + 0.628*PA*_3_. In practical applications, cost savings can be realized by having only one camera position. Therefore, when the required accuracy is low, estimating mass and volume simultaneously from only the dimensional information of the side view or the projected area information of the top view is recommended.

## Introduction

*Rosa roxburghii* belongs to the genus *Rosa* of the family *Rosaceae* and is mainly produced in the southwestern part of China, such as Yunnan, Guizhou, and Sichuan^1^. *Rosa roxburghii* fruits are rich in trace elements and various vitamins and have high nutritional and health value^2^. In 2022, 140,000 hectares of *Rosa roxburghii* were planted in Guizhou^3^. Global fruit and vegetable production reached 2.85 billion tons in 2019^4^. Therefore, sorting and grading equipment, processing and handling system design, and packaging and transportation machinery for agricultural products, including *Rosa roxburghii* fruit, are essential. Human grading of *Rosa roxburghii* fruits in an agricultural setting is inefficient and labor-intensive. Inconsistent grading results are due to the variable appearance of the fruit and the difficulty in identifying the associated physical characteristics. The mechanical grading operation helps to obtain materials with the same geometric properties, rationalize the use of fruits and vegetables with different classification characteristics, minimize the costs associated with packaging and transportation, and thus achieve the optimal packaging configuration^5^. It also allows consumers to select fruits of different sizes and masses, as consumers prefer fruits of the same shape.

Fruit characteristics such as dimension size, shape, color, texture, mass, and volume can be used as a standard basis for grading. Grading becomes complicated if the fruits are identical in appearance. This is because characteristics such as color, texture, and shape are difficult to define with standard values. Therefore, most scholars have used mass and volume as essential characteristics of mechanical grading, which are considered more economical and reliable^6^. In large production lines, calculating the mass and volume of each fruit unit is difficult^7^. Generally, only three dimensions of length, width, and height are measured, and formulas calculate the projected area and volume. Then, the estimative mass model is built based on these physical characteristics, such as grapefruit, sweet cherries, and dried tail fruits^8–10^. However, fewer studies have been conducted to develop volume estimation models based on these physical characteristics, which may be related to the different densities of fruits. Because most pulp and kernel have different material properties, some fruits even have cavities, which can lead to a weak correlation between fruit volume and other physical characteristics.

Most studies obtain dimensional information using measuring tools such as vernier calipers, leading to bottom efficiency and significant errors in the projected area calculated using dimensions^11–13^. With the improved camera resolution and the perfection of computer image processing methods, the accuracy of obtaining the physical characteristics of fruits by computer vision has been very high. It has been widely used in grading operations^14–17^. Computers replace human labor, automatically extract physical characteristics, make the fastest mass and volume estimations, control machinery for grading operations, and minimize errors^18,19^. Image processing technology is widely applied in various fields, such as robot navigation, aircraft navigation, medical scanning, industrial measurement, and agricultural harvesting and processing^20–23^. Researchers have given greater attention to the method of processing images by computer to build a mathematical model to determine the fruit’s morphology^24^. For example, Khoshnam et al. used image processing software that can directly obtain the diameters and projected areas of pomegranates in the three vertical directions. Based on the projected areas, they obtained a mass estimation model with a coefficient of determination (R^2^) up to 0.96^25^. Mansouri et al. obtained a mathematical model for optimal melon seed mass estimation by obtaining three-dimensional information (length, width, and height) of melon seeds from images^26^. However, when obtaining dimension information, it is often necessary to place the apparent dimension position (maximum or minimum diameter) of the fruit in the horizontal or vertical direction of the image, and the actual placement cannot be uniform.

Therefore, this paper presents a method for determining the dimensions of near-ellipsoidal fruits from images. The main research objective is to find the optimal estimation model for the mass and volume of *Rosa roxburghii* fruits based on the dimensional and projected area information of 2D images.

## Materials and methods

### Material acquisition

Newly harvested *Rosa roxburghii* fruits were picked and purchased from an orchard in Longli County, Guizhou Province, China. The experiments were performed after the harvested *Rosa roxburghii* fruits were carefully delivered to the laboratory and refrigerated at 8 ℃ for 24 h. Physical measurements were made on 60 random *Rosa roxburghii* fruits with no obvious surface defects or damage.

**Figure 1:**
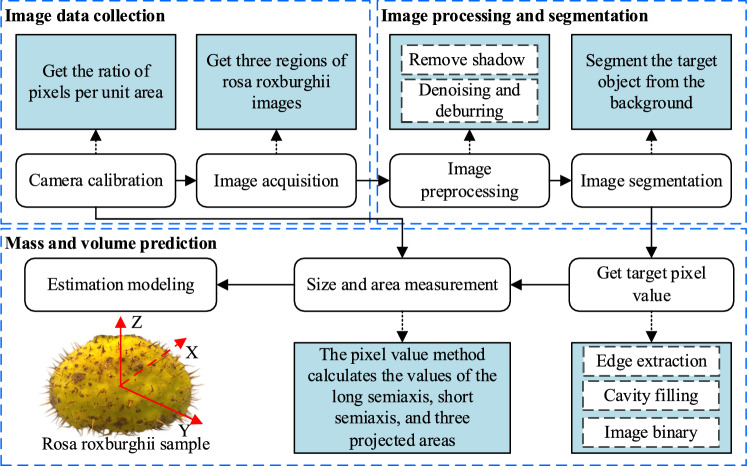
General process for image-based mass and volume estimation of *Rosa roxburghii* fruits.

Figure 1 shows the process of image-based estimation of *Rosa roxburghii* mass and volume. Before the image acquisition step, the volume and mass of the *Rosa roxburghii* samples need to be determined manually. Then, the ratio of pixel values per unit area needs to be obtained, and then pictures are taken along the three coordinate axes of the *Rosa roxburghii* in the figure. The mass (M) of each *Rosa roxburghii* fruit was measured on a digital balance with an accuracy of 0.01 g. The water displacement method measured the final volume (V), keeping the water temperature at 25 ℃^27^. Before selecting these 60 *Rosa roxburghii* fruits, we made a preliminary classification into three categories: large, medium and small. The parameter values of the 20 fruits selected for each category are shown in Table 1. The image processing and segmentation step is to reduce the noise interference of the picture, and the *Rosa roxburghii* picture is de-pricked so that the shape of the *Rosa roxburghii* fruit can be effectively segmented from the background^28^. With the above processing, dimensional and projected area measurements were determined to obtain an estimation model for volume and mass.

**Table 1:** Parameter values for three categories of *Rosa roxburghii* fruits.

Classification	Number	Parameter	Max	Min	Mean	Standard deviation
Large	20	Mass/g	29.76	20.17	24.88	2.96
		Volume/ml	30	19	23.95	3.51
Medium	20	Mass//g	18.76	14.78	15.99	1.04
		Volume/ml	20	12	16.45	2.38
Small	20	Mass//g	14.46	10.73	13.15	1.11
		Volume/ml	17	9	12.47	2.03

### Image processing

#### Image acquisition

**Figure 2:**
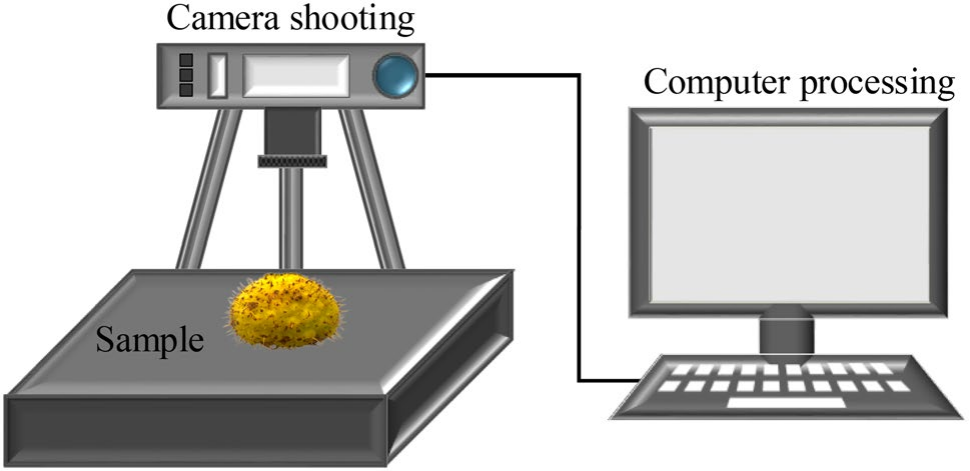
Image acquisition device and equipment.

Image acquisition was carried out on a horizontal experimental bench (Fig. 2), and the images of the *Rosa roxburghii* were obtained by a Canon G1 X camera whose basic parameters were set to a focal length of f = 35 mm and a sensor size of 18.7 × 14.0 mm. The dimensions extracted from the image are measured in pixels. Therefore, obtaining the number of pixel points per unit length and per unit area is necessary, which are converted to actual dimensions and areas utilizing pre-determined constant value ratios. For the camera used in this work, any object captured from a constant distance of H = 500 mm was measured to have 0.016 pixels per 1 mm^2^ and 0.126 pixels per 1 mm.

#### Image preprocessing

**Figure 3:**
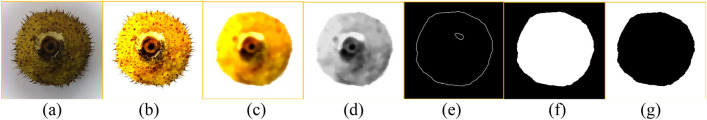
(**a**) Original image (**b**) adjust saturation and contrast of the image (**c**) de-pricking and denoising (**d**) gray-level thresholding for further de-shading (**e**) edge detection and generating voids (**f**) fill a void (**g**) final binary image.

Image preprocessing is necessary to improve image quality, mainly to reduce the impact of impurities and illumination in complex environments and to prepare for the subsequent segmentation of the complete *Rosa roxburghii* fruit contour. In this paper, the processing method of Javidan et al. is used in the preprocessing of *Rosa roxburghii* images, and also the method is effective in filling the voids produced by the images^29,30^. Due to the special external characteristics of the *Rosa roxburghii* fruits, and the ultimate goal of this paper, obtaining binary images after removing the prickly, we have made some changes in the image processing steps to apply to our study. The original image (Fig. 3a) produces shadows due to lighting effects, which can seriously affect the segmentation effect, resulting in shadows being segmented along with the target object. In Fig. 3b, we have adjusted the saturation and contrast to increase the color difference between the fruit and the background, making subsequent de-pricking and denoising processing easier. In Fig. 3c, a 5 × 5 median filter is used for de-pricking and denoising. Shadows can be removed by adjusting the gray value threshold (Fig. 3d gray image with appropriate threshold). In this paper, the Canny operator is used for edge detection to obtain clear edge lines and to facilitate the filling of voids, and boundary tracking is used to connect the boundaries (Fig. 3e). Figure 3f shows the filled binary image. Figure 3g shows the complete *Rosa roxburghii* binary image. The *Rosa roxburghii* contours and pixel-valued surfaces are kept as full as possible.

#### *Rosa roxburghii* image segmentation

**Figure 4:**
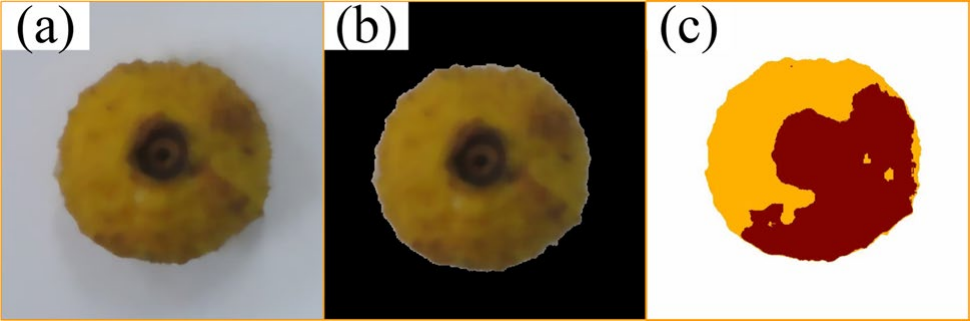
(**a**) Preprocessed image (**b**) HSV color space segmentation (**c**) K-means clustering segmentation.

Image segmentation is used to extract the shape of the *Rosa roxburghii* fruits from the background to facilitate the acquisition of a complete binary image^31^. The first one is HSV color space segmentation since RGB color space describes the combination of red, green, and blue in an image and is more sensitive to changes in light. Therefore, HSV color space can be used. Separate the fruit from the background by setting thresholds for hue and saturation. The second is K-means clustering segmentation, where the fruit’s color is significantly different from the background, and more than two different clusters are set to segment the fruit. As shown in Fig. 4b,c, these two methods are used to segment the preprocessed *Rosa roxburghii* fruit image (Fig. 4a). However, HSV color space segmentation in different lighting environments, setting the saturation threshold is different, the definition of the edge of *Rosa roxburghii* fruit is not very accurate, and finding the optimal threshold is time-consuming. Therefore, we use K-means clustering segmentation and set the same threshold for large-scale segmentation processing.

### Determination and analysis of physical characteristics

**Figure 5:**
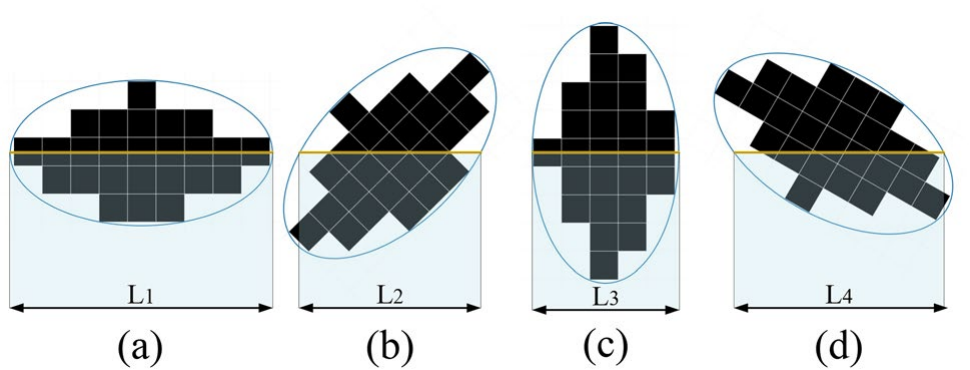
Measuring dimensions by the approximate ellipse method.

Some scholars place apparent size positions (such as maximum and minimum diameters) in the horizontal or vertical direction when making image measurements. This is unreasonable, and it cannot be guaranteed that the positions of the apparent size are entirely appropriate. Therefore, we use the approximate axisymmetric method. Each *Rosa roxburghii* fruit is approximated as an ellipsoid. Then the three projection surfaces are compared as an ellipsoid, and the most significant half-long axis and half-short axis in the three ellipsoids are the half-long axis (a) and half-short axis (b) of *Rosa roxburghii* fruit. The principle is shown in Fig. 5. The ellipse is oriented along the horizontal line, and only the number of pixel values in the center position is maximum, so the horizontal line segment is maximum. By traversing each row of 01 numbers in the direction of the horizontal line of the target binary image, the horizontal line segment whose ellipse has the largest number of pixel values at that position is found. When photographed, *Rosa roxburghii* fruits are not guaranteed to have a long or short axis on the horizontal. So, we need to rotate the image several times, with each rotation traversing to measure its maximum value. As shown in Fig. 5a–d, the lengths of the four line segments are different. The maximum of the largest value of all the rotated images is measured (L1), which is the length of the pixel value for the long axis, and the minimum of the largest value (L3), which is the length of the pixel value for the short axis. By calibrating the camera, we can easily know the pixel values per unit area and unit length, which can be converted to get the actual length.

Projected area measurements can be calculated from dimensional information, but the formulas assume that the contours are regular geometric shapes^10^. However, most fruits, such as sweet potatoes and mangoes, have irregular contours, and their projected areas can be obtained from images^7,32^. First, calculate the number of 01 in the binary image of *Rosa roxburghii* fruits after segmentation, and then through the pre-measured pixel value ratio per unit area, we can find the projected area of the three regions of *Rosa roxburghii*, which are perpendicular to each other, where the projected area of *Rosa roxburghii* indicates ${PA}_{1}$, ${PA}_{2}$, and ${PA}_{3}$. Among them, ${PA}_{1}$ and ${PA}_{2}$ are side view surfaces along the X-axis and Y-axis, respectively, and ${PA}_{3}$ is a top view surface along the Z-axis.The calculated criterion projected area (*CPA*) formula is then as follows:

1 $$CPA=\frac{P{A}_{1}+P{A}_{2}+P{A}_{3}}{3}.$$

**Figure 6:**
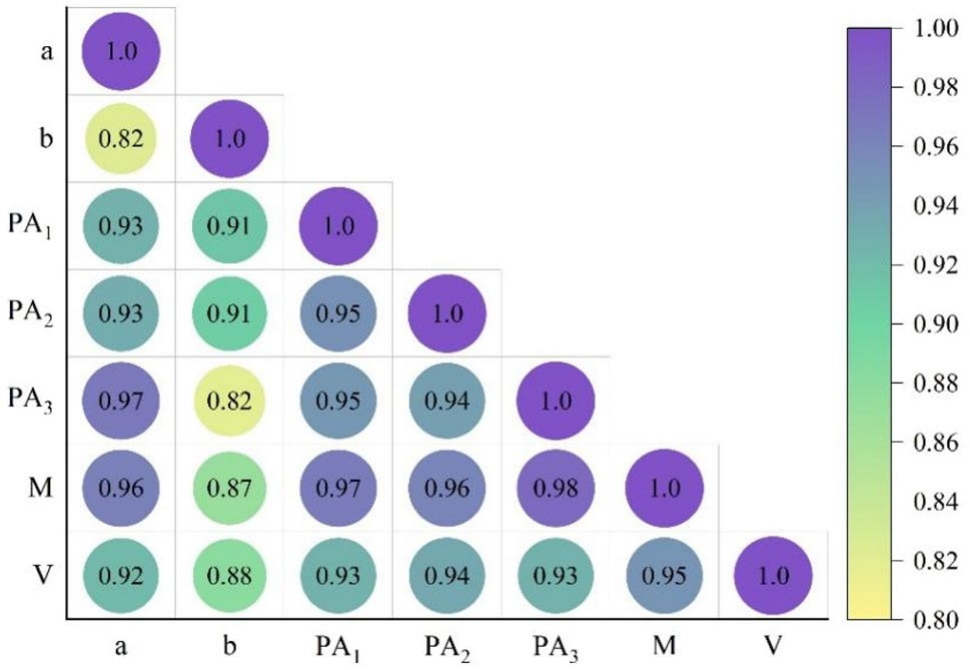
Relationship between the determination of physical characteristics of *Rosa roxburghii* fruits. a is the half-length axis and b is the half-short axis. *PA* projected area. *CPA* criterion projected area.

Table 2 shows the maximum, minimum, and average values of the dimensions and projected areas determined in the above ways. A digital balance measures the mass, and the volume is measured by the water displacement method. The table also shows that the difference between the areas measured from ${PA}_{1}$ and ${PA}_{2}$ is not significant. There is severe interoperability because these two surfaces are perpendicular to each other in the side view and have similar characteristics. The relationship between each physical characteristic’s measured properties (correlation coefficients) is shown in Fig. 6. The correlation coefficients between the measured length of the half-short axis (b) and the input data were low (0.82–0.91), while the correlation coefficients for the other parameters ranged from 0.92 to 0.98. This information will help in the development and design of grading equipment^33^.

**Table 2:** Data on physical characteristics of *Rosa roxburghii* fruits.

Parameter	Unit	Max	Min	Mean ± standard deviation
a	mm	25.39	15.49	19.72 ± 2.24
b	mm	17.71	11.53	14.76 ± 1.38
${PA}_{1}$	cm^2^	13.82	6.39	9.49 ± 1.85
${PA}_{2}$	cm^2^	13.41	6.15	9.37 ± 1.85
${PA}_{3}$	cm^2^	18.60	8.08	12.24 ± 2.72
$CPA$	cm^2^	14.97	7.05	10.37 ± 2.10
M (mass)	g	10.15	29.89	18.01 ± 5.38
V (volume)	ml	30	9	17.64 ± 5.52

a is the half-length axis and b is the half-short axis.

*PA* projected area, *CPA* criterion projected area.

### Evaluation of image measurement methods

**Figure 7:**
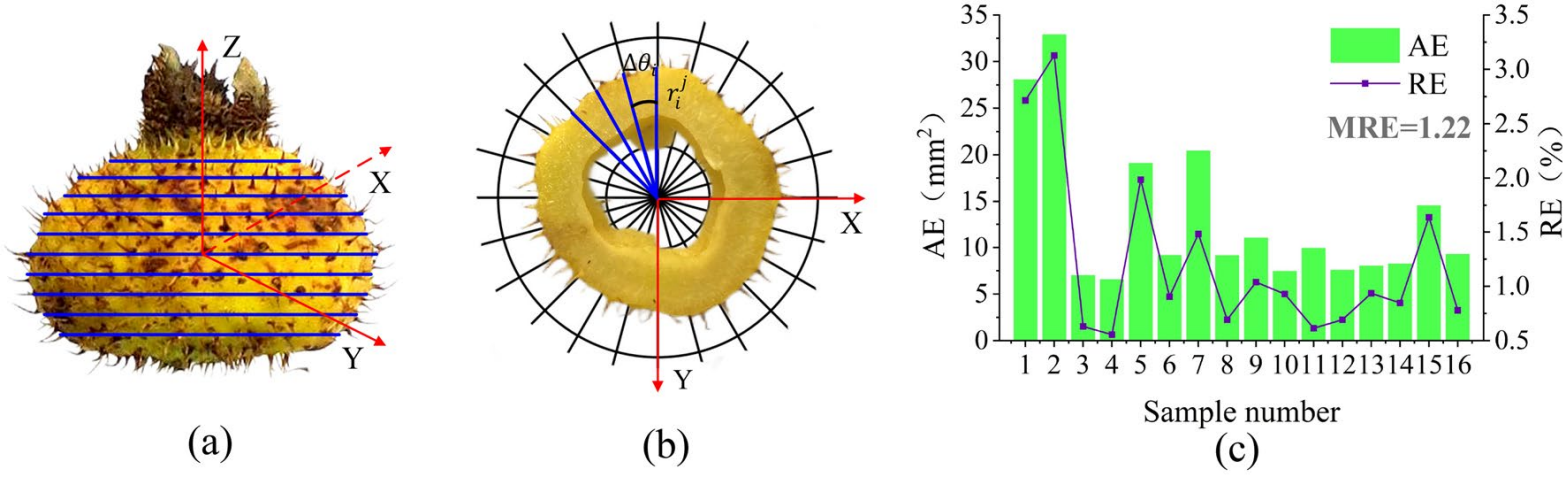
(**a**) Schematic of slicing (**b**) slice-and-integrate method of measuring area (**c**) error evaluation.

The dimensions and projected areas were obtained under the same shooting conditions. Therefore, it is only necessary to verify the consistency of the area obtained by the pixel value method with the actual area. We first used the pixel value method to photograph the maximum top-view surface of 16 *Rosa roxburghii* fruits, extracted the pixel values, and converted them to area. Then, we measured the actual area using the slice-and-integrate method^34^. The principle is shown in Fig. 7a. Along the vertical direction of the Z-axis, horizontal to the XY-plane, the *Rosa roxburghii* fruit is continuously sliced. Each slice is calculated in the coordinate paper, as shown in Fig. 7b. The actual area of the *Rosa roxburghii* fruit is the integral area of the largest slice. The integration formula is as follows:

2 $${A}^{j}=\sum_{i=0}^{n}\frac{\Delta {\theta }_{i}\pi {\left({r}_{i}^{j}\right)}^{2}}{360},$$

where $\Delta {\theta }_{i}$ is the step angle, ${r}_{i}^{j}$ is the radius of the step angle, and *n* is the total number of steps.

Figure 7c shows the absolute error (AE) and relative error (RE) between the 16 areas obtained by the pixel value method and the actual area. The mean relative error (MRE) was used as the main criterion for evaluating the accuracy of the image measurements, as in Eq. (3), which has an MRE of 1.22%. The results show that the pixel value method can better reflect the actual projected area and dimensions of *Rosa roxburghii* fruits.

3 $$\text{MRE}=\frac{1}{n}\sum_{i=1}^{n}\frac{\left|pv-mv\right|}{mv}\times 100\text{\%},$$

where $pv$ is the image measurement, $mv$ is the slice-and-integrate method measurement, $n$ is the total number of measurements.

### Mass and volume estimation methods

#### Dimension-based estimative modeling

For mass and volume estimation, univariate regression models (linear, quadratic, exponential, power) were used. The dependent equations are as in Eqs. (4)–(7)^35^.

4 $$Y={k}_{1}+{k}_{2}X,$$

5 $$Y={k}_{1}+{k}_{2}X+{k}_{3}{X}^{2},$$

6 $$Y={k}_{1}{e}^{{k}_{2}\text{X}},$$

7 $$Y={k}_{1}{X}^{{k}_{2}},$$

where *Y* is the dependent variable which can be mass *M* (g) or volume *V* (ml), “*X*” is the physical parameters related to the estimated object including a, b, ${PA}_{1}$,${PA}_{2}$, ${PA}_{3}$, and CPA. ${k}_{1}$, ${k}_{2}$ and ${k}_{3}$ are fitting constants.

The a and b, obtained from the images, are used as independent variables to complete the mass and volume modeling using the mathematical model in Eqs. (4)–(7). In addition, the multiple regression model is fitted based on the two independent variables, with equations as in Eqs. (8) and (9)^36^.

8 $$M={k}_{1}+{k}_{2}a+{k}_{3}b,$$

9 $$V={k}_{1}+{k}_{2}a+{k}_{3}b,$$

where ${k}_{1}$, ${k}_{2}$ and ${k}_{3}$ are regression constants.

#### Projected area-based estimative modeling

The three projected areas obtained from the images were used as independent variables to complete the mass and volume modeling using the mathematical models in Eqs. (4)–(7) . In addition, the multiple regression model is fitted based on the three independent variables with equations as in Eqs. (10) and (11).

10 $$M={k}_{1}+{k}_{2}{PA}_{1}+{k}_{3}{PA}_{2}+{k}_{4}{PA}_{3},$$

11 $$V={k}_{1}+{k}_{2}{PA}_{1}+{k}_{3}{PA}_{2}+{k}_{4}{PA}_{3},$$

where ${k}_{1}$, ${k}_{2}$, ${k}_{3}$ and ${k}_{4}$ are regression constants.

#### Geometric volume-based estimative modeling

In the third classification, we first need to assume that the *Rosa roxburghii* fruits are ellipsoidal and parabolic in shape based on the measured dimensions of the fruits (half-long axis a and half-short axis b), with equations as in Eqs. (12) and (13). The volume is obtained as the independent variable by these two equations. Then, the mass and volume modeling is completed according to the mathematical model in Eqs. (4)–(7).

12 $${V}_{ellip}=4\uppi {a}^{2}b/3,$$

13 $${V}_{parab}=4\uppi a{b}^{2}/3.$$

## Plant guideline statement

We confirm that all the experimental research and field studies on plants (either cultivated or wild), including the collection of plant material, complied with relevant institutional, national, and international guidelines and legislation. The *Rosa roxburghii* in this study is not a United Nations endangered species. All of the material is owned by the authors and/or no permissions are required. The plant specimens for this study are deposited in the Chinese Virtual Herbarium at https://www.cvh.ac.cn/spms/detail.php?id=f7e5c506. The collection barcode is GZTM0066333 and the identifier is Weike Jiang.

## Results and discussions

### Dimension-based mass and volume models

Analytical software such as Matlab fits the model and analyzes the sample data. The coefficient of determination (R^2^), chi-square (χ^2^), and root mean square error (RMSE) were chosen as criteria for evaluating the regression model’s applicability. The model with larger R^2^, smaller χ^2^, and RMSE was selected as appropriate.

**Figure 8:**
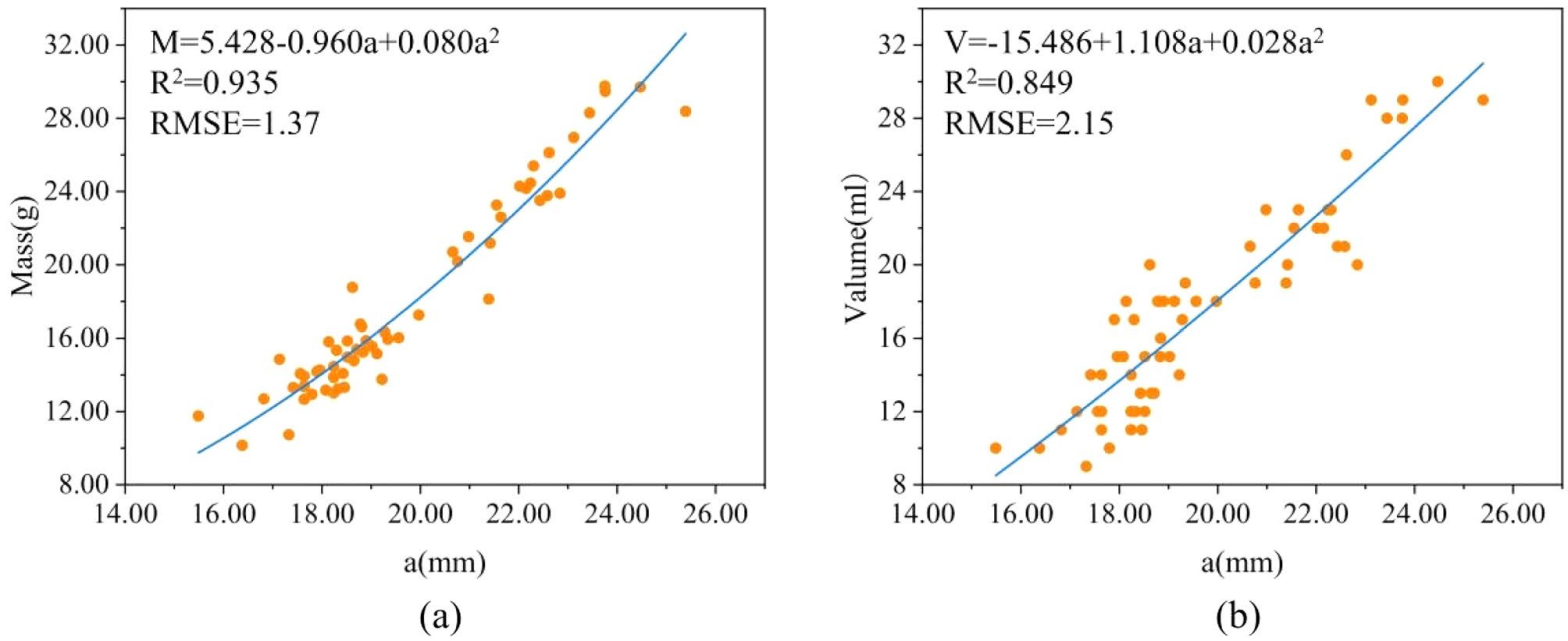
(**a**) Estimated mass quadratic model based on half-length axis (**a**) (**b**) estimated volume quadratic model based on half-length (**a**). R^2^ is coefficient of determination. RMSE root mean square error.

Table 3 shows the estimation models for mass and volume based on dimensions. As in Eqs. (14) and (15), the multiple regression model based on two dimensions (a and b) had the largest R^2^, the smallest χ^2^ and RMSE, and was estimated to be optimal for mass (R^2^ = 0.948, χ^2^ = 5.38, RMSE = 1.22) and volume (R^2^ = 0.896, χ^2^ = 11.63, RMSE = 1.78). As shown in Fig. 8, the quadratic model based on an estimate of mass and volume is better in terms of the single-factor estimation of mass and volume.

**Table 3:** Dimension-based estimation models for mass and volume.

Model	Constants			R^2^	χ^2^	RMSE
	*k*_1_	*k*_2_	*k*_3_			
Mass prediction model						
$M={k}_{1}+{k}_{2}a$	− 27.485	2.307	–	0.929	7.26	1.43
$M={k}_{1}+{k}_{2}b$	− 31.952	3.384	–	0.759	23.69	2.63
$M={k}_{1}+{k}_{2}a+{k}_{3}{a}^{2}$	5.428	− 0.960	0.080	0.935	6.26	1.37
$M={k}_{1}+{k}_{2}b+{k}_{3}{b}^{2}$	44.588	− 6.947	0.346	0.782	19.89	2.50
$M={k}_{1}{e}^{{k}_{2}\text{b}}$	1.168	0.183	–	0.779	20.28	2.52
$M={k}_{1}{e}^{{k}_{2}\text{b}}$	1.168	0.183	–	0.779	20.28	2.52
$M={k}_{1}{a}^{{k}_{2}}$	0.012	2.446	–	0.935	6.29	1.37
$M={k}_{1}{b}^{{k}_{2}}$	0.009	2.805	–	0.778	20.95	2.53
$M={k}_{1}+{k}_{2}a+{k}_{3}b$	− 31.970	1.829	0.941	0.948	5.38	1.22
Volume prediction model						
$V={k}_{1}+{k}_{2}a$	− 27.203	2.271	–	0.848	17.48	2.15
$V={k}_{1}+{k}_{2}b$	− 34.525	3.530	–	0.778	24.80	2.60
$V={k}_{1}+{k}_{2}a+{k}_{3}{a}^{2}$	− 15.486	1.108	0.028	0.849	17.33	2.15
$V={k}_{1}+{k}_{2}b+{k}_{3}{b}^{2}$	3.190	− 1.561	0.170	0.775	23.43	2.57
$V={k}_{1}{e}^{{k}_{2}\text{a}}$	1.691	0.117	–	0.839	18.51	2.22
$V={k}_{1}{e}^{{k}_{2}\text{b}}$	1.031	0.190	–	0.776	23.78	2.61
$V={k}_{1}{a}^{{k}_{2}}$	0.012	2.443	–	0.846	17.59	2.16
$V={k}_{1}{b}^{{k}_{2}}$	0.007	2.920	–	0.782	23.38	2.58
$V={k}_{1}+{k}_{2}a+{k}_{3}b$	− 34.539	1.490	1.540	0.896	11.63	1.78

a is the half-length axis and b is the half-short axis. R^2^ is coefficient of determination. χ^2^ is chi-square.

*RMSE* root mean square error.

14 $$M=-31.970+1.829a+0.941b,$$

15 $$V=-34.539+1.490a+1.540b.$$

### Projected area-based mass and volume models

**Figure 9:**
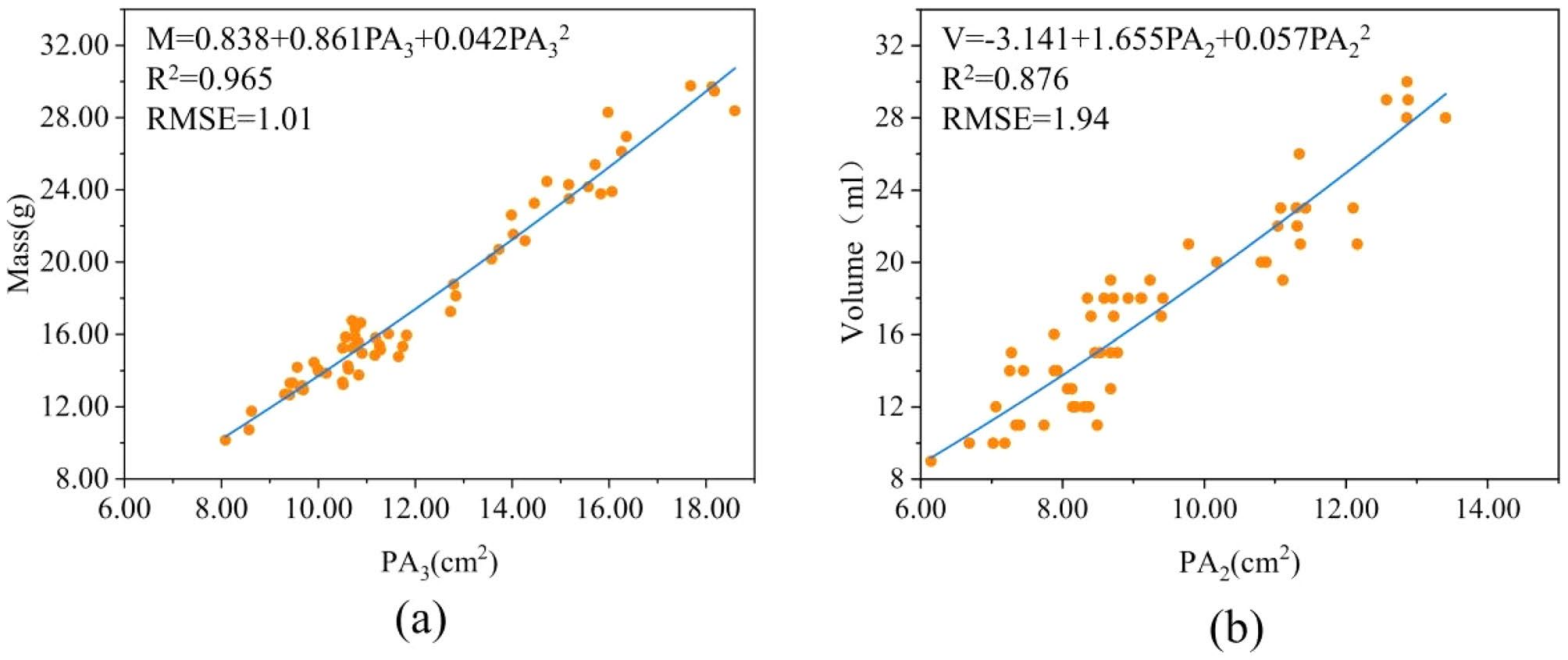
(**a**) Estimated quadratic model of mass based on ${PA}_{3}$ (**b**) estimated quadratic model of volume based on ${PA}_{2}$. *PA* projected area. R^2^ is coefficient of determination. RMSE root mean square error.

Table 4 shows the estimation models based on the projected area. As in Eq. (16), the quadratic model based on *CPA* estimates the optimal mass, with R^2^ (0.981) being the largest and χ^2^ (1.82) and RMSE (0.73) being the smallest. As in Eq. (17), the quadratic model based on three dimensions (${PA}_{1}$, ${PA}_{2}$, and ${PA}_{3}$) estimates the optimal volume, with R^2^ (0.898) being the largest and χ^2^ (11.83) and RMSE (1.76) being the smallest. The model is based on CPA and three dimensions, and all three facets must be measured simultaneously. Therefore, when considering the single factor estimation model as in Fig. 9, the quadratic model based on ${PA}_{3}$ is better in estimating mass. The quadratic model based on ${PA}_{2}$ estimates the volume better.

**Table 4:** Projected area-based estimation models for mass and volume.

Model	Constants				R^2^	χ^2^	RMSE
	*k*_1_	*k*_2_	*k*_3_	*k*_4_			
Mass prediction model							
$M={k}_{1}+{k}_{2}{PA}_{1}$	− 8.512	2.793	–	–	0.934	6.94	1.38
$M={k}_{1}+{k}_{2}{PA}_{2}$	− 7.987	2.774	–	–	0.923	7.49	1.48
$M={k}_{1}+{k}_{2}{PA}_{3}$	− 5.671	1.934	–	–	0.964	3.39	1.02
$M={k}_{1}+{k}_{2}CPA$	− 8.109	2.519	–	–	0.978	2.17	0.79
$M={k}_{1}+{k}_{2}{PA}_{1}+{k}_{3}{{PA}_{1}}^{2}$	4.864	0.036	0.137	–	0.941	5.92	1.30
$M={k}_{1}+{k}_{2}{PA}_{2}+{k}_{3}{{PA}_{2}}^{2}$	7.561	− 0.400	0.166	–	0.933	6.28	1.39
$M={k}_{1}+{k}_{2}{PA}_{3}+{k}_{3}{{PA}_{3}}^{2}$	0.838	0.861	0.042	–	0.965	3.24	1.01
$M={k}_{1}+{k}_{2}CPA+{k}_{3}{CPA}^{2}$	0.280	0.940	0.071	–	0.981	1.82	0.73
$M={k}_{1}{e}^{{k}_{2}{PA}_{1}}$	4.475	0.143	–	–	0.938	6.07	1.33
$M={k}_{1}{e}^{{k}_{2}{PA}_{2}}$	4.479	0.145	–	–	0.932	6.31	1.39
$M={k}_{1}{e}^{{k}_{2}{PA}_{3}}$	5.336	0.096	–	–	0.954	4.01	1.15
$M={k}_{1}{e}^{{k}_{2}CPA}$	4.620	0.128	–	–	0.976	2.25	0.82
$M={k}_{1}{{PA}_{1}}^{{k}_{2}}$	0.691	1.445	–	–	0.939	6.26	1.32
$M={k}_{1}{{PA}_{2}}^{{k}_{2}}$	0.696	1.448	–	–	0.929	6.80	1.42
$M={k}_{1}{{PA}_{3}}^{{k}_{2}}$	0.635	1.332	–	–	0.965	3.22	1.01
$M={k}_{1}{CPA}^{{k}_{2}}$	0.620	1.435	–	–	0.981	1.86	0.74
$M={k}_{1}+{k}_{2}{PA}_{1}+{k}_{3}{PA}_{2}+{k}_{4}{PA}_{3}$	− 7.659	0.714	0.560	1.114	0.980	27.75	2.83
Volume prediction model							
$V={k}_{1}+{k}_{2}{PA}_{1}$	− 8.618	2.759	–	–	0.858	17.89	2.08
$V={k}_{1}+{k}_{2}{PA}_{2}$	− 8.500	2.783	–	–	0.875	13.96	1.95
$V={k}_{1}+{k}_{2}{PA}_{3}$	− 5.463	1.882	–	–	0.860	15.87	2.07
$V={k}_{1}+{k}_{2}CPA$	− 8.170	2.483	–	–	0.896	12.18	1.78
$V={k}_{1}+{k}_{2}{PA}_{1}+{k}_{3}{{PA}_{1}}^{2}$	0.333	0.915	0.092	–	0.861	17.49	2.06
$V={k}_{1}+{k}_{2}{PA}_{2}+{k}_{3}{{PA}_{2}}^{2}$	− 3.141	1.655	0.057	–	0.876	13.86	1.94
$V={k}_{1}+{k}_{2}{PA}_{3}+{k}_{3}{{PA}_{3}}^{2}$	− 6.582	2.060	− 0.007	–	0.860	15.85	2.07
$V={k}_{1}+{k}_{2}CPA+{k}_{3}{CPA}^{2}$	− 7.181	2.297	0.008	–	0.896	12.19	1.78
$V={k}_{1}{e}^{{k}_{2}{PA}_{1}}$	4.327	0.144	–	–	0.857	18.03	2.08
$V={k}_{1}{e}^{{k}_{2}{PA}_{2}}$	4.301	0.146	–	–	0.870	14.85	1.99
$V={k}_{1}{e}^{{k}_{2}{PA}_{3}}$	5.262	0.096	–	–	0.845	17.93	2.17
$V={k}_{1}{e}^{{k}_{2}CPA}$	4.502	0.128	–	–	0.886	13.93	1.87
$V={k}_{1}{{PA}_{1}}^{{k}_{2}}$	0.626	1.476	–	–	0.861	17.51	2.06
$V={k}_{1}{{PA}_{2}}^{{k}_{2}}$	0.648	1.469	–	–	0.876	13.89	1.94
$V={k}_{1}{{PA}_{3}}^{{k}_{2}}$	0.690	1.289	–	–	0.858	16.11	2.08
$V={k}_{1}{CPA}^{{k}_{2}}$	0.602	1.438	–	–	0.895	12.44	1.79
$V={k}_{1}+{k}_{2}{PA}_{1}+{k}_{3}{PA}_{2}+{k}_{4}{PA}_{3}$	− 8.467	0.657	1.294	0.628	0.898	11.83	1.76

*R*^*2*^ coefficient of determination, *χ*^*2*^ chi-square, *PA* projected area, *CPA* criterion projected area, *RMSE* root mean square error.

16 $$M=0.280+0.940CPA+0.071{CPA}^{2},$$

17 $$V=-8.467+0.657{PA}_{1}+1.294{PA}_{2}+0.628{PA}_{3}.$$

### Geometric volume-based mass and volume models

**Figure 10:**
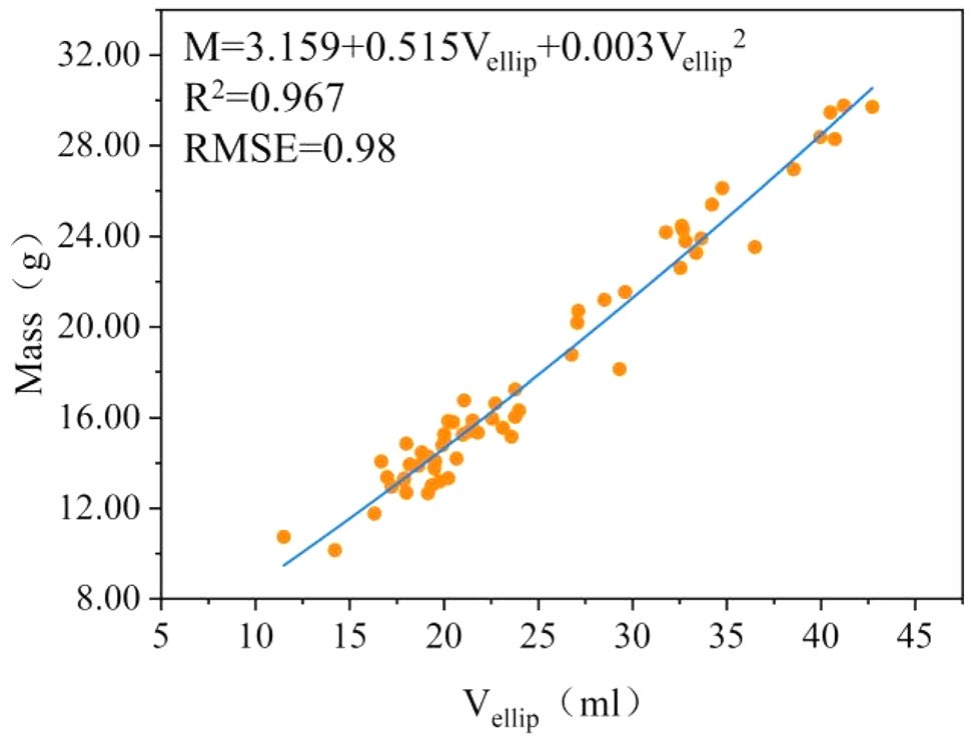
Estimated quadratic model of mass based on ellipsoidal volume (${V}_{ellip}$). R^2^ is coefficient of determination. *RMSE* root mean square error.

Table 5 shows the estimated models based on geometric volumes. Among the mass models, the quadratic model based on the ellipsoid method (Eq. 18) is the most appropriate. Figure 10 shows the fitted plot, with R^2^ (0.967) being the largest, and χ^2^ (3.34) and RMSE (0.98) being the smallest. In the volume model, the correlation between the volume measured by the ellipsoid method and the actual volume of the fruit was better (R^2^ = 20.902). Still, its RMSE (7.95) was too large, and it did not allow for an accurate estimation of the fruit volume. The RMSE of the volume measured by the parabolic method (2.11) was smaller, so the volume calculated by the parabolic method was closer to the actual volume of the *Rosa roxburghii* fruit from the analysis of the computed error.

**Table 5:** Geometric volume-based estimation models for mass and volume.

Model	Constants			R^2^	χ^2^	RMSE
	*k*_1_	*k*_2_	*k*_3_			
Mass prediction model						
$M={k}_{1}+{k}_{2}V$	1.809	0.921	–	0.901	9.90	1.68
$M={k}_{1}+{k}_{2}{V}_{ellip}$	1.085	0.679	–	0.966	3.54	0.99
$M={k}_{1}+{k}_{2}{V}_{parab}$	0.462	0.944	–	0.913	8.35	1.58
$M={k}_{1}+{k}_{2}V+{k}_{3}{V}^{2}$	7.128	0.310	0.016	0.911	8.32	1.59
$M={k}_{1}+{k}_{2}{V}_{ellip}+{k}_{3}{{V}_{ellip}}^{2}$	3.159	0.515	0.003	0.967	3.34	0.98
$M={k}_{1}+{k}_{2}{V}_{parab}+{k}_{3}{{V}_{parab}}^{2}$	2.981	0.677	0.006	0.914	8.04	1.57
$M={k}_{1}{e}^{{k}_{2}V}$	7.572	0.047	–	0.907	8.46	1.63
$M={k}_{1}{e}^{{k}_{2}{V}_{ellip}}$	7.476	0.034	–	0.957	4.04	1.10
$M={k}_{1}{e}^{{k}_{2}{V}_{parab}}$	7.227	0.047	–	0.906	8.51	1.64
$M={k}_{1}{V}^{{k}_{2}}$	1.302	0.917	–	0.898	10.38	1.71
$M={k}_{1}{{V}_{ellip}}^{{k}_{2}}$	0.858	0.947	–	0.965	3.64	1.00
$M={k}_{1}{{V}_{parab}}^{{k}_{2}}$	1.024	0.981	–	0.912	4.40	1.58
Volume prediction model						
${V}_{ellip}=4\uppi {a}^{2}b/3$	–	–	–	0.902	206.46	7.95
${V}_{parab}=4\uppi a{b}^{2}/3$	–	–	–	0.887	17.41	2.11

${V}_{ellip}$ is ellipsoidal volume, ${V}_{ellip}$ is parabolic volume, *R2* is coefficient of determination, χ^2^ is chi-square, *RMSE* root mean square error.

18 $$M=3.159+0.515{V}_{ellip}+0.003{{V}_{ellip}}^{2}.$$

### Evaluation of three estimation models

In Tables 3, 4, and 5, we developed estimation models for *Rosa roxburghii* mass and volume based on dimensions, projected area, and geometric volume. Among the mass estimation models established based on these three classifications, the optimal one is the quadratic model based on the criteria projected area (*CPA*), as in Eq. (16), R^2^ = 0.981, and RMSE = 0.73.

Among the volume estimation models based on these three classifications, the optimal is the multiple regression model based on three dimensions (${PA}_{1}$, ${PA}_{2}$, and ${PA}_{3}$), as in Eq. (17), R^2^ = 0.898 and RMSE = 1.76. Based on other similar studies on fruits, it was found that the multiple regression model and the mathematical model based on the criteria projected area had a high correlation and better reflected the relationship between the physical characteristics measured and the mass and volume of the fruits. Related species such as apple ber (*Ziziphus mauritiana* L.) had multiple regression models predicting mass and volume with R^2^ of 0.935 and 0.950, respectively^33^. The multiple regression model for elephant apple (*Dillenia indica* L.) had an R^2^ of 0.935 for predicting mass^18^.

The optimal model for estimating mass and volume above requires multiple factors to be involved in the calculation simultaneously, which requires three camera positions to capture. However, in practical applications, only one camera position can save costs. In addition, when sorting large quantities, using physical features extracted from the same image to estimate mass and volume simultaneously enables fast computer processing^25^. Therefore, in cases where the impact on estimation accuracy is not significant, measuring both mass and volume is recommended based solely on physical information obtained from a single image. When we photographed the three faces of *Rosa roxburghii*, the two side view surfaces based on the X and Y axes measured highly similar areas. Therefore, if the side view camera position based on *Rosa roxburghii*, it is recommended that a multiple regression model based on the two dimensions (a and b) be estimated for mass (R^2^ = 0.948, RMSE = 1.22) and volume (R^2^ = 0.896, RMSE = 1.78). If the top view camera position is based on the Z-axis of *Rosa roxburghii*, it is recommended to estimate the mass (R^2^ = 0.965, RMSE = 1.01) and volume (R^2^ = 0.860, RMSE = 2.07) based on the quadratic model of ${PA}_{3}$.

**Figure 11:**
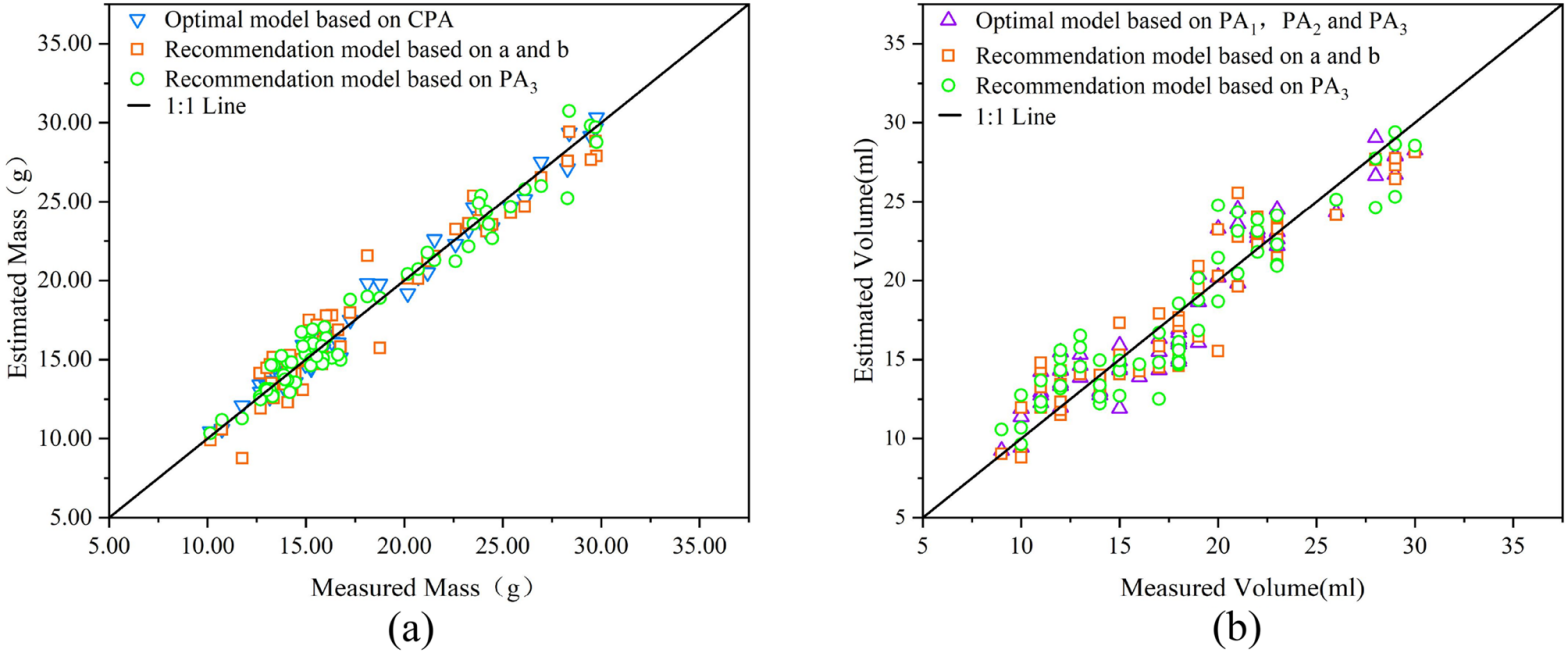
Comparison of recommended and optimal estimates of mass and volume with measured values: (**a**) quadratic model based on *CPA*, multiple regression model based on a and b, quadratic model based on ${PA}_{3}$ (**b**) multiple regression model based on ${PA}_{1}$, ${PA}_{2}$, and ${PA}_{3}$, multiple regression model based on a and b, quadratic model based on ${PA}_{3}$. a is the half-length axis and b is the half-short axis. *PA* projected area. *CPA* criterion projected area.

We compare the optimal estimates in the model with the recommended estimates and also compare the accuracy (φ) of each estimation model, as in Eq. (19). Figure 11a shows the comparison between the estimated mass and the mass measured by the scale. The two recommended mass estimation models are not very different from the optimal estimation model. The optimal estimation model based on *CPA* has an accuracy of 99.27%, the recommended estimation model based on a and b has an accuracy of 98.78%, and the recommended estimation model based on ${PA}_{3}$ has an accuracy of 98.99%. Figure 11b shows the comparison between the estimated volume and the volume measured by the water displacement method. The two recommended volume estimation models are similar to the optimal estimation model. the optimal estimation model based on the three dimensions (${PA}_{1}$, ${PA}_{2}$, and ${PA}_{3}$) has an accuracy of 98.24%. The recommended estimation model based on a and b has an accuracy of 98.22%, and the recommended estimation model based on ${PA}_{3}$ has an accuracy of 97.93%. This result indicates that estimating mass and volume from physical features obtained from a single camera position is feasible.

19 $$ \varphi = \frac{100 - RMSE}{{100}} \times 100\% . $$

## Conclusions

In this paper, we propose an approach to estimate the mass and volume of *Rosa roxburghii* by obtaining the dimensions and projected area of the fruit based on a two-dimensional image. When measuring the dimensional information, we propose to approximate *Rosa roxburghii* as an ellipsoid to obtain any placement position to measure its half-long and half-short axes. In this paper, the reliability of the image measurement method was also verified at the same time, and the mean relative error (MRE) was 1.22% compared with the results of the actual measurements carried out by the slicing method, which proves that the method can better reflect the dimensional information and projected area of *Rosa roxburghii*. In the mass and volume estimation models, the dependent variable parameters of the best estimation model must be obtained simultaneously from three camera positions. The best estimation model for mass is the quadratic model based on the criterion projected area (the average of the three projected areas), R^2^ = 0.981, and the equation is $M=0.280+0.940CPA+0.071{CPA}^{2}$. The best estimation model for volume is the multiple regression model based on the three projected areas, R^2^ = 0.898, and the equation is $V=-8.467+0.657{PA}_{1}+1.294{PA}_{2}+0.628{PA}_{3}$. From an economic point of view, using only one camera position can save costs, and it is recommended to estimate both mass (R^2^ = 0.948) and volume (R^2^ = 0.896) based on the dimensional information of the side view plane (multiple regression model for a and b). Alternatively, a quadratic model based on the projected area of the top view surface is estimated simultaneously for mass (R^2^ = 0.965) and volume (R^2^ = 0.860). Therefore, the image measurement method in this study can be effectively applied to measure the physical characteristics of *Rosa roxburghii*, and the obtained estimation model can also be used to improve *Rosa roxburghii* processing machines.

## Data Availability

All the data generated/analyzed during the study are available with the corresponding author on reasonable request.
